# Multiple Evidences for Association between Cognitive Impairment and Dysglycemia in Parkinson’s Disease: Implications for Clinical Practice

**DOI:** 10.3389/fnagi.2017.00355

**Published:** 2017-11-03

**Authors:** Liu Yang, Zhilin Chen, Bo Li, Meihua Wang, Lijia Yu, Ying Wan, Jing Gan, Yu Zhang, Zhenguo Liu, Xijin Wang

**Affiliations:** ^1^Department of Neurology, Xinhua Hospital, Shanghai Jiao Tong University School of Medicine, Shanghai, China; ^2^Department of Endocrinology, Xinhua Hospital, Shanghai Jiao Tong University School of Medicine, Shanghai, China

**Keywords:** cognitive impairment, dysglycemia, Parkinson’s disease, glycosylated hemoglobin A1c, risk factors

## Abstract

**Background and purpose:** It remains unclear about the etiopathogenesis of cognitive impairment (CI) in Parkinson’s disease (PD). Since diabetes mellitus (DM) has been shown to be associated with CI in several diseases, we examined the association between CI and dysglycemia in PD.

**Methods:** Enrolled PD patients completed a series of clinical and neuropsychological assessments. Motor symptoms were determined by Hohen-Yahr staging (H-Y staging) and Unified Parkinson’s Disease Rating Scale – motor score (UPDRS-III). Neuropsychological functions were evaluated by the Mini Mental State Examination (MMSE), the Montreal Cognitive Assessment (MoCA), and the Hamilton Anxiety and Depression Scales. Moreover, fasting glucose, fasting insulin, glycosylated hemoglobin A1c (HbA1c) and oral glucose tolerance test were performed to assess glucose metabolism.

**Results:** MoCA and MMSE scores in PD patients with DM group (PD-DM) were significantly lower than those in PD patients without DM group (PD-nDM). Consistently, PD-DM group showed significantly higher constituent ratio of CI than PD-nDM group. In addition, MoCA scores in HbA1c ≥ 6.5% group and HbA1c ≥ 7% group were significantly lower than those in the corresponding control groups. MoCA score in IR ≥ 3 group was significantly lower than that in IR < 3 group. Furthermore, MoCA score was negatively correlated with H-Y staging, HbA1c and insulin resistance, respectively. Finally, regression analysis indicated that H-Y staging and HbA1c ≥ 7% were independent risk factors of CI in PD.

**Conclusion:** CI may be tightly associated with dysglycemia in, at least partially, PD patients. Importantly, H-Y staging and HbA1c ≥ 7%, two independent risk factors of CI in PD, may serve as key biomarkers in future PD clinical practice.

## Introduction

Parkinson’s disease (PD) has been recognized as a multisystem neurodegenerative disorder with typical motor symptoms, including static tremor, bradykinesia, rigidity, postural instability, and gait difficulty. Although movement symptoms are the main clinical characteristics of PD, increasing evidence has shown that PD patients often experience a series of non-motor symptoms, including cognitive impairment (CI), dysfunction of autonomic nervous system, and behavioral disturbances.

Cognitive dysfunction is one of the most devastating and common non-motor symptoms of PD. There are approximately 20–40% of patients suffering from CI in the early stage of PD (Aarsland et al., 2010). The average incidence of mild CI has shown to be 26.7% (range, 18.9–38.2%) in PD (Litvan et al., 2011). More than 75–80% of the patients will eventually develop to dementia (Litvan et al., 2011). The cumulative incidence of dementia in PD approached to 80% in a community-based study (Hely et al., 2008). A prospective study suggested that individuals with PD had a three–suxfold higher risk of developing dementia than people without PD at the same age (Aarsland et al., 2001). PD severely impacts on the patients’ life quality, imposing a huge burden on the patients, their family and society. CI makes this hard situation even worse in PD patients.

According to a nationally cross-sectional survey in China, the overall prevalence of diabetes mellitus (DM) was estimated to be 11.6% in the Chinese adult population (Xu et al., 2013). Diabetes is frequently accompanied with a variety of complications. Many studies have shown a relationship between diabetes and cognitive decline, and this correlation is particularly significant in patients over 60 years old (Xu et al., 2009). Several epidemiological studies have suggested that diabetes is associated with the development of PD. For example, diabetes has been shown to be one risk factor for the occurrence of PD (Cereda et al., 2011). Diabetes may aggravate movement symptoms of PD patients (Cereda et al., 2012; Kotagal et al., 2013).

However, little is known about the association between CI and dysglycemia in PD. Therefore, in the present study, we investigated the association between cognitive function and dysglycemia in PD with the following aims: to compare clinical characteristics of Parkinson’s disease patients with (PD-DM) or without (PD-nDM) diabetes mellitus, to compare cognitive function at different levels of HbA1c and insulin resistance in Parkinson’s disease patients, and finally to investigate risk factors of CI in Parkinson’s disease.

## Materials and Methods

### Participants

A total of 282 PD patients were recruited in the department of Neurology at Xinhua hospital affiliated to Shanghai Jiao Tong University from October 2013 to October 2016. All the participants were examined by a movement disorder neurologist and met United Kingdom PD Society Brain Bank Research Center clinical criteria, and completed a series of clinical and neuropsychological assessment at the “on” status. The exclusion criteria were (1) patients with parkinsonism syndrome secondary to drugs, cerebrovascular disease, encephalitis, metabolic disease except diabetes, malignant tumor, carbon monoxide poisoning, or any other neurological and psychiatric disorders; (2) patients had prior or planned neurosurgical treatment (e.g., deep brain stimulation); (3) patients had anticholinergic medications (e.g., trihexyphenidyl, memantine); other medical or neurological causes of CI (e.g., seizures, strokes, head trauma, and neurosyphilis); (4) patients suffered from a serious chronic physical disease or diseases that affect glucose metabolism (e.g., cardiac insufficiency, renal failure, thyroid dysfunction) and; (5) patients with fasting glucose <3.9 mmol/L; (6) patients were unable to complete the questionnaire. This study was carried out in accordance with the recommendations of the Ethics Committee of Xinhua Hospital affiliated to Shanghai Jiao Tong University School of Medicine with written informed consent from all patients or their legal guardians. All patients or their legal guardians were given the informed consent form in accordance with the Declaration of Helsinki. The protocol was approved by the Ethics Committee of Xinhua Hospital affiliated to Shanghai Jiao Tong University School of Medicine.

### Clinical Data Collection

The detailed demographics characteristics of study participants included age, gender, body mass index (BMI), disease-related variables, medications, and educational background (measured as the total number of school years). All the participants underwent Hohen-Yahr staging (H-Y staging) and Unified Parkinson’s Disease Rating Scale – motor score (UPDRS-III) to evaluate motor symptoms. All participants underwent neuropsychological assessment by an experienced neuropsychologist. General cognitive function was measured using the Mini Mental State Examination (MMSE), which has a maximum score of 30. Dementia severity was assessed using Montreal Cognitive Assessment (MoCA), which includes the following seven domains: visuospatial/executive (5′), naming (3′), delayed memory (5′), attention (6′), language (3′), abstraction (2′), and orientation (6′). If the informed years of education were ≤12 years, then 1′ was added to correct the bias for education level. Patients with MoCA scores <26′ were considered to have CI (Dalrymple-Alford et al., 2010; Litvan et al., 2012; Marras et al., 2013). Emotion evaluations were based on Hamilton Anxiety Scale (HAMA, 14 items) and Hamilton Depression Scale (HAMD, 24 items) (Hamilton, 1967; Xiao et al., 2011; Kandiah et al., 2014).

### Biochemical Tests

Biochemical tests for fasting glucose, HbA1c, fasting insulin and oral glucose tolerance test (OGTT) were performed in 119 participants. Procedure for 2h-OGTT required administration of 75 g oral glucose load within a 5-min period, and 2-h postprandial glucose was subsequently drawn at 120 min, timed from the beginning of the glucose load (Hu et al., 2004; Cukierman-Yaffe et al., 2009; Bosco et al., 2012; Cereda et al., 2012; Yaffe et al., 2012). A fasting glucose ≥6.1 mmol/L was considered as high fasting glucose (Hu et al., 2004; Cukierman-Yaffe et al., 2009; Cereda et al., 2012; Yaffe et al., 2012). Patients were considered to have an impaired glucose tolerance (IGT) if 2-h postprandial glucose was between 7.8 and 11.1 mmol/L (Hu et al., 2004; Cukierman-Yaffe et al., 2009; Cereda et al., 2012; Yaffe et al., 2012). Insulin resistance (IR) was calculated by the homoeostasis model assessment (HOMA) formula [HOMA-Index: Basal Glucose Plasma (mmol/L) × Basal Insulin Plasma (mU/L)/22.5] (Matthews et al., 1985; Bosco et al., 2011, 2012). Patients were considered to have insulin resistance if their HOMA-Indexes ≥3 (Matthews et al., 1985). DM was defined by one of the following criteria: participants reported diabetes history, discharge diagnosis of DM, a tested fasting glucose level higher than 7.0 mmol/L, or a 2-h postprandial glucose level higher than 11.1 mmol/L (Boon et al., 1997; Hu et al., 2004; Cukierman-Yaffe et al., 2009; Cereda et al., 2012; Yaffe et al., 2012).

### Statistical Analyses

All statistical procedures were conducted using Statistical Package for the Social Sciences (SPSS) version 20.0 for Windows. Comparison between groups was performed using a two-sample *t*-test (normal distribution) or Mann–Whitney *U*-test (abnormal distribution) as appropriate. Categorical variables were examined using the Chi-square test. Correlation tests were performed using spearman correlation analysis, and the statistically significant indicators were included in the logistic regression analysis. Statistical significance was set at *p* < 0.05.

## Results

### Clinical Characteristics of PD-DM and PD-nDM Patients

**FIGURE 1:**
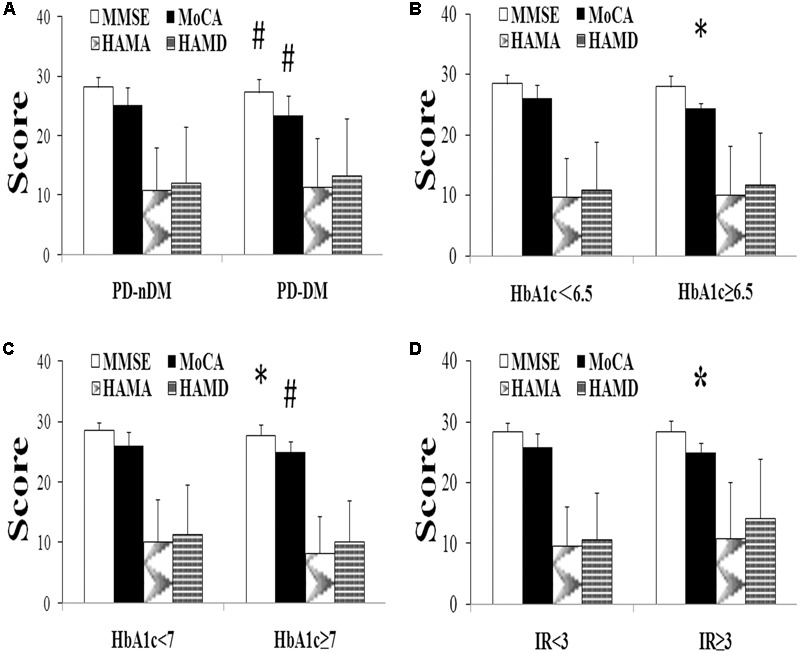
Neuropsychological assessment in Parkinson’s disease (PD). **(A)** Comparison of neuropsychological function of PD patients with (PD-DM, *n* = 85) or without (PD-nDM, *n* = 197) diabetes mellitus. **(B)** Comparison of neuropsychological function of PD patients with HbA1c < 6.5 (*n* = 90) or HbA1c ≥ 6.5 (*n* = 29). **(C)** Comparison of neuropsychological function of PD patients with HbA1c < 7 (*n* = 95) or HbA1c ≥ 7 (*n* = 24). **(D)** Comparison of neuropsychological function of PD patients with IR < 3 (*n* = 104) or IR ≥ 3 (*n* = 15). HbA1c, glycosylated hemoglobin A1c; IR, insulin resistance; MMSE, Mini Mental State Examination; MoCA, Montreal Cognitive Assessment; HAMA, Hamilton Anxiety Scale; HAMD, Hamilton Depression Scale. ^∗^*p* < 0.05, compared with the corresponding control group; #*p* < 0.01, compared with the corresponding control group.

A total of 282 PD patients were recruited in this study, 85 of them with DM (30.1%) (PD-DM) and 197 of them without diabetes (69.9%) (PD-nDM). As shown in **Table 1**, there was no significant difference in age, gender, education, levodopa equivalent dosage, various types of drug ratio, onset age, duration, onset form, UPDRS-III, H-Y staging, HAMA score, or HAMD score between PD-DM and PD-nDM groups. Body mass index (BMI) in PD-DM group was significantly higher than that in PD-nDM group (23.17 ± 1.57 vs. 22.56 ± 2.28 kg/m^2^, *p* = 0.010) (**Table 1**). Interestingly, MoCA score (23.35 ± 3.41 vs. 25.12 ± 2.99, *p* = 0.000) and MMSE score (27.32 ± 2.28 vs. 28.18 ± 1.74, *p* = 0.003) in PD-DM group were significantly lower than those in PD-nDM group (**Figure 1A**). In addition, PD-DM group showed significantly higher constituent ratio of CI compared to PD-nDM group (x2 = 21.400, *p* = 0.000) (**Table 2**).

**Table 1:** Clinical characteristics of PD-DM and PD-nDM patients.

	PD-nDM	PD-DM	
	(*n* = 197)	(*n* = 85)	*P*
Gender (male: number/percentage)	108/54.8%	44/51.8%	0.697^c^
Age (year)	69.10 ± 8.21	70.79 ± 7.63	0.121^a^
Duration (year)	5.02 ± 5.48	5.05 ± 5.22	0.430^b^
Onset age (year)	64.08 ± 9.46	65.74 ± 8.12	0.171^a^
Current smoking (number/percentage)	65/33.0%	23/27.1%	0.332^c^
Body mass index (BMI, kg/m^2^)	22.56 ± 2.28	23.17 ± 1.57	**0.010^a^**
Degree of education (year)	11.62 ± 3.36	10.92 ± 4.29	0.064^b^
**Form of onset**			
Tremor (number/percentage)	114/57.9%	42/49.4%	0.195^c^
Dominant side involved (number/percentage)	103/52.3%	50/58.8%	0.362^c^
**Drugs**			
Levodopa intake (number/percentage)	177/89.8%	80/94.1%	0.268^c^
levodopa equivalent dosage (mg/day)	469.54 ± 289.15	540.15 ± 319.34	0.092^a^
levodopa dosage (mg/day)	409.14 ± 258.46	475.29 ± 295.97	0.128^a^
DRA (number/percentage)	76/38.6%	36/42.4%	0.597^c^
MAOBI (number/percentage)	38/19.3%	17/20.0%	0.890^c^
COMTI (number/percentage)	5/2.5%	5/5.9%	0.291^c^
**Movement symptoms**			
UPDRS-III	21.91 ± 13.85	23.78 ± 14.41	0.279^b^
H-Y staging			0.126^c^
0–1 (number/percentage)	65/33.0%	18/21.2%	
1.5–2 (number/percentage)	73/37.1%	39/45.9%	
2.5–3 (number/percentage)	59/29.9%	28/32.9%	

*PD-nDM, Parkinson’s disease patients without diabetes mellitus; PD-DM, Parkinson’s disease patients with diabetes mellitus; DRA, dopamine receptor agnoists; MAOBI, Monoamine oxidase type B inhibitors; COMTI, Catechol-*O*-methyltransferase inhibitors; UPDRS-III, Unified Parkinson’s Disease Rating Scale motor score; H-Y staging, Hohen-Yahr staging; ^a^two-sample *t*- test; ^b^Mann–Whitney *U*-test; ^c^Chi-square test.*

**Table 2:** Comparison of constituent ratio of cognitive impairment (CI) in PD-nDM and PD-DM patients.

	PD-nDM	PD-DM	*P*
	(*n* = 197)	(*n* = 85)	
PD-nCI	110/55.8%	22/25.9%	**0.000^c^**
PD-CI	87/44.2%	63/74.1%	

*PD-nDM, PD patients without diabetes mellitus; PD-DM, PD patients with diabetes mellitus; PD-nCI, PD patients without cognitive impairment; PD-CI, PD patients with cognitive impairment. ^c^Chi-square test.*

### Comparison of Cognitive Function at Different Levels of HbA1c and Insulin Resistance in Parkinson’s Disease Patients

In our current study, HbA1c and HOMA-index were used as parameters to evaluate diabetes control and insulin resistance in PD, respectively. As shown in **Figures 1B,C**, MoCA scores in HbA1c ≥ 6.5% group (24.41 ± 0.91 vs. 26.02 ± 2.21, *p* = 0.016) and HbA1c ≥ 7% group (24.96 ± 1.85 vs. 26.03 ± 2.21, *p* = 0.007) were significantly lower than those in the corresponding control groups. Furthermore, MoCA score in IR ≥ 3 group was significantly lower than that in IR < 3 group (24.93 ± 1.58 vs. 25.94 ± 2.23, *p* = 0.049) (**Figure 1D**). In addition, none of HbA1c ≥ 6.5%, HbA1c ≥ 7% or IR ≥ 3 groups showed significant difference in age, gender, education, levodopa equivalent dosage, drug ratio, onset age, duration, onset form, UPDRS-III, H-Y staging, MMSE score, HAMA score, or HAMD score compared with the corresponding control groups (**Tables 3–5**).

**Table 3:** Clinical characteristics of HbA1c < 6.5 and HbA1c ≥ 6.5 groups in PD.

	HbA1c < 6.5	HbA1c ≥ 6.5	*^P^*
	(*n* = 90)	(*n* = 29)	
Gender (male: number/percentage)	51/56.7%	15/51.7%	0.672^c^
Age (year)	70.47 ± 7.79	68.69 ± 7.31	0.267^a^
Duration (year)	4.15 ± 4.18	4.59 ± 3.21	0.181^b^
Onset year (year)	66.32 ± 8.80	64.10 ± 8.55	0.162^a^
Current smoking (number/percentage)	30/33.3%	6/20.7%	0.249^c^
Body mass index (BMI, Kg/m^2^)	22.67 ± 1.34	23.28 ± 1.67	0.070^a^
Degree of education (year)	11.90 ± 2.53	12.00 ± 2.36	0.795^b^
**Form of onset**			
Tremor (number/percentage)	42/46.7%	14/48.3%	1.000^c^
Dominant side involved (number/percentage)	49/54.4%	17/58.6%	0.830^c^
**Drugs**			
Levodopa intake (number/percentage)	81/90.0%	29/100%	0.111^c^
levodopa equivalent dosage (mg/day)	490.42 ± 295.83	474.14 ± 211.45	0.970^a^
levodopa dosage (mg/day)	436.67 ± 270.64	394.83 ± 173.38	0.143^a^
DRA (number/percentage)	38/42.2%	16/55.2%	0.284^c^
MAOBI (number/percentage)	17/18.9%	5/17.2%	1.000^c^
COMTI (number/percentage)	3/3.3%	1/3.4%	1.000^c^
**Movement symptoms**			
UPDRS-III	20.66 ± 12.06	19.93 ± 10.22	0.995^b^
H&Y			0.861^c^
0–1 (number/percentage)	32/35.6%	9/31.0%	
1.5–2 (number/percentage)	37/41.1%	12/41.4%	
2.5–3 (number/percentage)	21/23.3%	8/27.6%	

*HbA1c, glycosylated hemoglobin A1c; DRA, dopamine receptor agnoists; MAOBI, Monoamine oxidase type B inhibitors; COMTI, Catechol-*O*-methyltransferase inhibitors; UPDRS-III, Unified Parkinson’s Disease Rating Scale motor score, H-Y staging, Hohen-Yahr staging; ^a^two-sample *t*- test; ^b^Mann–Whitney *U*-test; ^c^Chi-square test.*

**Table 4:** Clinical characteristics of HbA1c < 7 and HbA1c ≥ 7 groups in PD.

	HbA1c < 7	HbA1c ≥ 7	*^P^*
	(*n* = 95)	(*n* = 24)	
Gender (male: number/percentage)	55/57.9%	11/45.8%	0.360^c^
Age (year)	70.41 ± 7.71	68.54 ± 7.54	0.239^a^
Duration (year)	4.17 ± 3.05	4.28 ± 4.17	0.578^b^
Onset year (year)	66.13 ± 8.64	64.38 ± 9.23	0.303^a^
Current smoking (number/percentage)	31/32.6%	5/20.8%	0.326^c^
Body mass index (BMI, Kg/m^2^)	22.73 ± 1.37	23.18 ± 1.67	0.156^a^
Degree of education (year)	12.01 ± 2.53	11.58 ± 2.26	0.509^b^
**Form of onset**			
Tremor (number/percentage)	44/46.3%	12/50.0%	0.821^c^
Dominant side involved (number/percentage)	51/53.7%	15/62.5%	0.496^c^
**Drugs**			
levodopa equivalent dosage (mg/day)	503.29 ± 298.44	419.79 ± 153.40	0.286^a^
levodopa dosage (mg/day)	441.05 ± 268.17	368.75 ± 153.09	0.370^a^
DRA (number/percentage)	43/45.3%	11/45.8%	1.000^c^
MAOBI (number/percentage)	19/20.0%	3/12.5%	0.560^c^
COMTI (number/percentage)	4/4.2%	0	0.582^c^
**Movement symptoms**			
UPDRS-III	20.64 ± 11.79	19.83 ± 11.03	0.827^b^
H&Y			0.840^c^
0–1 (number/percentage)	33/34.7%	8/33.3%	
1.5–2 (number/percentage)	40/42.1%	9/37.5%	
2.5–3 (number/percentage)	22/23.2%	7/29.2%	

*HbA1c, glycosylated hemoglobin A1c; DRA, dopamine receptor agnoists; MAOBI, Monoamine oxidase type B inhibitors; COMTI, Catechol-*O*-methyltransferase inhibitors; UPDRS-III, Unified Parkinson’s Disease Rating Scale motor score; H-Y staging, Hohen-Yahr staging; ^a^two-sample *t*- test; ^b^Mann–Whitney *U*-test; ^c^Chi-square test.*

**Table 5:** Clinical characteristics of IR < 3 and IR ≥ 3 groups in PD.

	IR < 3	IR ≥ 3	*^P^*
	(*n* = 104)	(*n* = 15)	
Gender (male: number/percentage)	58/55.8%	8/53.3%	0.859^c^
Age (year)	70.23 ± 7.54	68.67 ± 7.79	0.532^a^
Duration (year)	4.18 ± 4.08	4.73 ± 3.04	0.215^b^
Onset year (year)	66.04 ± 8.53	63.93 ± 10.31	0.374^a^
Current smoking (number/percentage)	33/31.7%	3/20.0%	0.394^c^
Body mass index (BMI, Kg/m^2^)	22.73 ± 1.42	23.18 ± 1.67	0.578^a^
Degree of education (year)	11.93 ± 2.56	11.87 ± 1.92	0.922^b^
**Form of onset**			
Tremor (number/percentage)	49/47.1%	7/46.7%	1.000^c^
Dominant side involved (number/percentage)	57/54.8%	9/60.0%	0.786^c^
**Drugs**			
Levodopa equivalent dosage (mg/day)	480.65 ± 280.49	526.67 ± 255.73	0.482^a^
Levodopa dosage (mg/day)	424.52 ± 257.32	440.00 ± 202.84	0.275^a^
DRA (number/percentage)	48/46.2%	6/11.1%	0.784^c^
MAOBI (number/percentage)	18/17.3%	4/26.7%	0.475^c^
COMTI (number/percentage)	4/3.8%	0	1.000^c^
**Movement symptoms**			
UPDRS-III	19.97 ± 11.37	24.00 ± 12.96	0.220^b^
H&Y			0.647^c^
0–1 (number/percentage)	37/35.6%	4/26.7%	
1.5–2 (number/percentage)	41/39.4%	8/53.3%	
2.5–3 (number/percentage)	26/25.0%	3/20.0%	

*IR, insulin resistance; DRA, dopamine receptor agnoists; MAOBI, Monoamine oxidase type B inhibitors; COMTI, Catechol-*O*-methyltransferase inhibitors; UPDRS-III, Unified Parkinson’s Disease Rating Scale motor score; H-Y staging, Hohen-Yahr staging; ^a^two-sample *t*- test; ^b^Mann–Whitney *U*-test; ^c^Chi-square test.*

### Risk Factors of Cognitive Impairment in Parkinson’s Disease

As shown in **Table 6**, MoCA score was negatively correlated with H-Y staging (*r* = -0.243, *p* = 0.008), HbA1c (*r* = -0.207, *p* = 0.024), and insulin resistance (*r* = -0.498, *p* = 0.000). However, MMSE score had no correlation with either H-Y staging or glucose metabolic indicators (**Table 6**). To identify the risk factors of CI, logistic regression analysis was conducted, and our data indicated that H-Y staging (OR = 1.844, 95% confidence interval:1.162 ∼ 2.927, *p* = 0.009) and HbA1c ≥ 7% (OR = 4.253, 95% confidence interval:1.596 ∼ 11.342, *p* = 0.004) were independent risk factors of CI in patients with PD (**Table 7**).

**Table 6:** Correlation of CI with H-Y staging, HbA1c and IR in PD.

	MMSE		MoCA	
	*r*	*P*	*r*	*P*
H-Y staging	-0.070	0.448^d^	-0.243	**0.008**^d^
HbA1c	-0.114	0.218^d^	-0.207	**0.024**^d^
IR	-0.027	0.772^d^	-0.498	**0.000**^d^

*MMSE, Mini Mental State Examination; MoCA, Montreal Cognitive Assessment; H-Y staging, Hohen-Yahr staging; HbA1c, serum glycosylated hemoglobin A1C; IR, insulin resistance; ^d^spearman correlation analysis.*

**Table 7:** Regression analysis on risk factors of CI in PD.

	*B*	*SE*	*P*	OR	95%CI
H-Y staging	0.612	0.236	**0.009**^e^	1.844	(1.162 ~ 2.927)
HbA1c ≥ 7%	1.448	0.500	**0.004**^e^	4.253	(1.596 ~ 11.342)
HbA1c ≥ 6.5%	0.044	1.059	0.967^e^	1.045	(0.131 ~ 8.329)
IR ≥ 3	0.508	0.768	0.509^e^	0.369	(0.777 ~ 14.279)

*H-Y staging, Hohen-Yahr staging; HbA1c, serum glycosylated hemoglobin A1C; IR, insulin resistance; ^e^logistic regression analysis.*

## Discussion

In this study, we investigated clinical features of PD patients with or without dysglycemia, examined risk factors of CI in PD, and explored the relationship between CI and dysglycemia in patients with PD.

Cognitive impairment is frequently found in PD, especially in the late stage of the disease (Hely et al., 2008; Aarsland et al., 2010; Litvan et al., 2011). It is well known that diabetes is one of the risk factors of cognitive dysfunction (Xu et al., 2009) in several diseases, but it remains unclear about the role of dysglycemia in CI of PD. In the present study, we observed that PD-DM patients had significant higher BMI compared to PD-nDM patients, which was in accordance with the clinical features of DM (Hu et al., 2004). There was no significant difference in onset form, dominant side involvement, levodopa equivalent dosage, doses of levodopa, UPDRS-III, or H-Y staging between PD-DM and PD-nDM patients. However, previous studies have suggested PD patients with diabetes may have worse motor symptoms compared to those without diabetes. A case-control study indicated that patients with PD and DM had higher motor scores, and needed larger doses of dopaminergic treatment compared to patients with PD only (Cereda et al., 2012). Another cohort study showed that diabetes could aggravate the movement symptoms of PD, especially gait disturbance and postural stability (Kotagal et al., 2013). The disparity between our results and other studies may be due to the difference of sample size and enrolled patient population. In line with the previous study (Profenno et al., 2010), our data showed significant differences in MMSE and MoCA scores were observed between PD-DM and PD-nDM patients. Moreover, the prevalence of cognitive dysfunction is significantly higher in PD-DM patients than that in PD-nDM patients, suggesting that diabetes may be one risk factor for cognitive dysfunction in, at least partially, PD patients. It has been shown that α- synuclein, amyloid-β (Aβ) peptides, and tau protein may be involved in the pathologic process of CI in PD (Weintraub et al., 2012; Kang et al., 2013). Laboratory studies suggested that CI was caused by aberrant glycation of α-synuclein, Aβ peptides and tau protein (Vicente Miranda et al., 2016), which was induced by glycation agent produced by dysglycemia (Sousa Silva et al., 2013). Insulin resistance may also lead to the abnormal regulation of tau, resulting in Aβ peptides deposition (Bosco et al., 2011). It is necessary to further investigate the specific pathological mechanism for CI in PD in our future research.

HbA1c, a commonly used parameter reflecting the mean glucose concentration during the past 8–12 weeks, is a superior indicator to assess long-term glycemic control (Miedema, 2005). Although several methods have been developed to diagnose human insulin resistance, the HOMA-index method has been recognized as the mostly employed approach in both clinical practice and epidemiological studies due to its simplicity (Matthews et al., 1985; Salgado et al., 2010). Therefore, in our current study, HbA1c and HOMA-index were used as parameters to evaluate diabetes control and insulin resistance in PD, respectively. We observed that MoCA scores in HbA1c ≥ 6.5%, HbA1c ≥ 7% and insulin resistance ≥3 groups were significantly lower than that in the corresponding control groups, suggesting CI is associated with the control situation of diabetes in, at least partially, PD patients. In our study, HbA1c ≥ 6.5, HbA1c ≥ 7 and IR ≥ 3 groups were significantly lower than the corresponding control groups, which is in agreement with actual clinical conditions and also observed in previous studies (Bohnen et al., 2014; Ong et al., 2017).

Furthermore, correlation analysis showed MoCA score was negatively correlated with H-Y staging, HbA1c and insulin resistance, respectively. However, MMSE score was not correlated with either H-Y staging or glucose metabolic indicators. The potential reason could be that MoCA has the higher sensitivity and specificity (Dalrymple-Alford et al., 2010), better evaluation reliability (Gill et al., 2008), and higher discriminant validity (Hoops et al., 2009) in the assessment of cognitive function in PD, whereas there is a ceiling effect of MMSE in cognitive evaluation (Hoops et al., 2009; Marras et al., 2013). Based on the previous studies (Dalrymple-Alford et al., 2010; Litvan et al., 2012; Marras et al., 2013), we set MoCA 26’ as a discriminator to differentiate CI, which was shown to be a convenience and reliable approach for clinicians to evaluate patients’ cognitive function.

Finally, logistic regression analysis showed that H-Y staging and HbA1c ≥ 7% were risk factors of CI in PD. The correlation of CI with H-Y staging is in accordance with the development characteristics of PD. With the progression of disease severity, cognitive dysfunction gradually deteriorated (Hely et al., 2008). Among all the glucose metabolic indicators, HbA1c ≥ 7% was a significant risk factor of CI in PD. Some large scale cross-sectional (Cukierman-Yaffe et al., 2009; Nguyen et al., 2010) and prospective studies (Kanaya et al., 2004; Yaffe et al., 2006, 2012) have indicated that lower score of MoCA is associated with higher level of HbA1c. The American Diabetes Association (ADA) proposed that HbA1c ≥ 6.5% can be used to diagnose DM, and keeping an HbA1c lower than 7% reduces the occurrence of diabetic microvascular lesion (Yaffe et al., 2012). Based on our study and previous studies, H-Y staging and HbA1c may serve as key parameters for controlling CI in PD clinical practice.

There are still some limitations in this study. First of all, the sample size of our study was relatively small, and the participants were mainly from Shanghai, China, therefore, our results may have a certain area or racial bias. Secondly, our study was a cross-sectional research, lacking of comparative support from prospective study. Thirdly, our study lacked cerebral vascular disease risk factors (such as drinking, hypertension, stroke) assessment, and there was no further analysis of the influence of diabetic medicines on patients’ cognitive function. Lastly, all the patients participated in our study had a H-Y staging 3 or less than 3, namely early stage PD patients. Therefore, our results may not apply to the whole PD group.

## Conclusion

Our study showed that CI may be tightly associated with dysglycemia in, at least partially, PD patients. Importantly, H-Y staging and HbA1c ≥7% may be independent risk factors of CI in PD, and may serve as key biomarkers in future PD clinical practice. Further studies are needed to investigate the specific mechanism for CI of PD.

## Author Contributions

XW conceived the project and designed the study. LYa, ZC, and BL contributed to participant recruitment, data collection and data analysis. MW and LYu contributed to participant recruitment and data collection. YW, JG, YZ, and ZL contributed to supervise the study. LYa, BL, and XW wrote the paper together.

## Conflict of Interest Statement

The authors declare that the research was conducted in the absence of any commercial or financial relationships that could be construed as a potential conflict of interest.
